# Creep and permeability evolution behavior of red sandstone containing a single fissure under a confining pressure of 30 MPa

**DOI:** 10.1038/s41598-020-58595-2

**Published:** 2020-02-05

**Authors:** Sheng-Qi Yang, Bo Hu

**Affiliations:** State Key Laboratory for Geomechanics and Deep Underground Engineering, School of Mechanics and Civil Engineering, China University of Mining and Technology, Xuzhou, 221116 P.R. China

**Keywords:** Geophysics, Civil engineering

## Abstract

The long-term deformation and permeability evolution with time are key issues for geo-engineering applications such as radioactive waste disposal. Rock permeability concurrent with deformation is significantly influenced by cracking. This study investigated the creep-permeability evolution behavior of red sandstone specimens containing a single fissure under a confining pressure of 30 MPa. First, the effects of stress ratio (SR) and fissure dip angle on the creep behavior of rock were investigated. The more loading/unloading cyclic numbers, the larger the irrecoverable axial deformation. The instant elastic strains and visco-elastic strains linearly increased with SR for both the intact and fissured specimens, whereas the instant plastic strains showed different results. The visco-plastic strains nonlinearly increased. For fissured and intact specimens, the creep strains and the steady-state creep rates nonlinearly increased as SR increased. The instantaneous strains, instant elastic strains, and visco-elastic strains slightly varied when the fissure dip angle was less than 45° but notably decreased with increasing fissure dip angle beyond 45°. However, the fissure dip angle had no obvious effects on the plastic and creep strains. Damage (*D*) was defined using the ratio of non-elastic strains to the total strain. *D* increased approximately linearly with SR, but the fissure dip angle had no obvious influences. Subsequently, the long-term strength (LTS) of the red sandstone was determined using two different methods. The LTS first decreased when the fissure dip angle increased from 0 to 45° but increased with increasing dip angle. The triaxial and creep failure modes were mainly shear along anti-wing cracks for the fissured specimens but shear failure occurred for the intact specimen. Moreover, the permeability of the fissured red sandstone was governed by SR and deformation or time. During the multi-step loading/unloading creep process, the permeability first decreased and then had a sudden rise when tertiary creep occurred.

## Introduction

Discontinuities (joints, fractures, cracks, and faults) widely exist in rock masses, which cause many difficulties for stability design in rock engineering. The discontinuities not only reduce the strength but also increase the deformation of the rock mass. Stress concentration in some regions occurs, helping the initiation of new discontinuities^1–4^. Therefore, it is challenging to predict the mechanical properties and failure patterns of the rock masses. Engineers and scientists are interested in the mechanical behavior of jointed rock mass^5,6^. However, it is hard to perform *in-situ* experiments, so various scholars have investigated the failure mechanisms of rock specimens or rock-like materials containing pre-existing flaws at indoor laboratories. Generally speaking, two main research areas focus on crack initiation patterns and on cracking modes under uniaxial compression and triaxial compression^4,7–12^. Previous investigations have promoted our understanding of crack initiation and coalescence processes for jointed rock masses under short-term loading. The results showed that wing cracks and secondary cracks are typical failure modes, although there were different failure patterns^13^.

In practice, long-term stability and support design of deep underground engineering should consider the creep mechanical behaviors of rock masses^14–19^. It is necessary to investigate the time-based deformation behavior and crack propagation of jointed rocks. Subcritical crack growth was one of the main reasons for the time-dependent behavior of brittle rock^20^. Laboratory tests have shown the effect of stress on induced anisotropy, which can lead to fatigue at cracks tips^21,22^. Brittle creep occurs under static fatigue conditions when the fracture toughness exceeds the stress intensity factor at crack tips^23,24^. Some researchers have concluded that the main mechanism of creeping in the rock is time-dependent cracking^25–27^. Kaiser and Morgenstern^28^ found that the visco-plasticity was apparent when the stress reached near the peak strength based on the time-dependent deformations of jointed coal. Hao *et al*.^29^ reported the results of brittle creep-relaxation experiments of rock, which may increase the understanding of the relationship between stress relaxation and time-to-failure and predict the time-dependent rupture and energetic release during the relaxation phase. Patton and Fletcher^30^ theoretically studied the time-dependent closure of a fracture with a rough surface under normal stress compression, and then, the constitutive equations were used to verify the response on two types of fractured layers. Fabre and Pellet^31^ presented the experimental results of argillaceous rocks with various fabric plane inclinations under different loading conditions and reported their time-dependent deformations. The microstructural analysis highlighted the granular creep. Zhao *et al*.^32^ compared the creep behaviors of intact and cracked limestones by a series of multi-step loading and unloading creep tests. However, most studies focused on the micro-flaws or subcritical cracks.

There have been reports on the creep behaviors of rock containing macro-cracks. The long-term strength (LTS) of jointed rock mass is also an essential parameter for stability evaluation. The time-dependent deformation of jointed rock mass plays a critical role in evaluating the long-term stability of rock mass engineering. Chen *et al*.^33^ proposed a damaged coupled visco-elastic-plastic model to simulate the time-dependent deformation of rock mass in the Shanxi YRDP project. A micromechanics-based model was developed to predict the creep deformation of hard rock and assess the long-term stability of the excavations^34^. Nara *et al*.^35^ obtained the crack velocity in andesite and high-strength and ultra-low permeability concrete in water and air environments. They estimated the LTS of the specimens, which provides a vital reference for assessing the long-term stability of the underground repository. Chandler^36^ determined the LTS of crystalline rocks, which was the stress corresponding to the reversal point at the volume strain curve. Generally, the LTS was less than the peak stress.

However, to date, the effects of macro fissure inclination angle on the long-term deformation and failure behavior of real hard rock contacting pre-existing fissure and its long-term permeability evolution during the creep process have not been extensively investigated. Therefore, to better understand the effects of a macro fissure on the elastic-viscoplastic deformation and LTS, a series of cyclic loading and unloading creep tests and gas permeability measurement of the intact and fissured rock containing a fissure with different inclinations are presented. Then, a new method will be presented to estimate the LTS of red sandstone.

## Fissured Rock Material and Testing Method

### Preparation of single-fissured red sandstone

**Figure 1:**
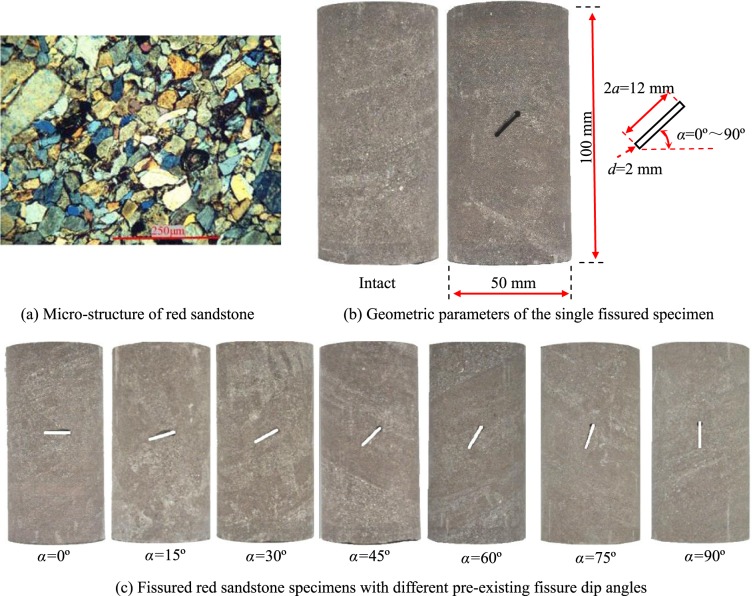
Tested red sandstone and geometry of pre-existing fissure.

Rock specimens of—red sandstone were collected from Rizhao City in Shandong Province, China. It has a classic and blocky structure as shown in Fig. 1a. Its dry density is 2402 kg/m^3^, and effective porosity is ~6.26–6.55%^37^. The mineral mainly comprises quartz and feldspar calcite, dolomite, hematite and clay minerals^38^. Cuboid blocked rock was first cut, and then the pre-existing fissures were produced using high-speed water jet technology to obtain fissured rock specimens with different fissure dip angles. Finally, cylindrical red sandstones with different fissure inclinations were drilled and polished. The height of the cylindrical fissured red sandstone was 50 × 100 mm and the length (2*a*) and width (2*d*) of the fissure were 12 and 2 mm, respectively. The dip angles of the fissure were from 0 to 90° (interval 15° and *α* is the dip between fissure and horizontal axial), as shown in Fig. 1b. The cylindrical red sandstones containing a single pre-existing fissure with different dip angles and intact specimen are presented in Fig. 1.

### Testing method

**Figure 2:**
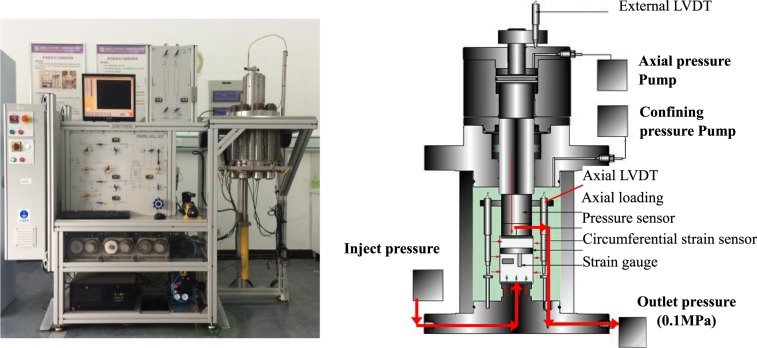
Rock triaxial rheological testing device^14^.

To investigate the influence of fissure dip angle on the creep mechanical behavior of red sandstone under high confining pressure and to study its gas permeability evolution during the creep deformation process, the triaxial compressive strength needs to be first determined before creep experiments. Specifically, traditional triaxial compressive tests under a confining pressure of 30 MPa on single-fissured red sandstones and the intact specimen were performed using a triaxial rock apparatus, as shown in Fig. 2 ^14^. First, the specimens were subjected to a hydrostatic pressure of 30 MPa at a rate of 5 MPa/min. Then, the specimens were subjected to axial loading at a rate of 0.02 mm/min until failure. Finally, the triaxial compressive strength (TCS) was obtained. Creep stress levels were set according to the TCS.

The multi-step loading-unloading method was used during creep experiments to study the elastic-visco-plastic creep deformations of the specimens. During creep loading and unloading stages, the loading rate and unloading rate were 6 MPa/min. Each loading stress steps lasted two days until accelerating creep occurred and the unloading stress steps (visco-elastic recovery) were only performed for a day. The axial and lateral displacements were recorded using two parallel linear variable differential transformers (LVDTs) in axial dimension and a circumferential displacement transducer, respectively. Nitrogen was used as a flow medium and the steady-state method was used to measure the gas permeability of the single-fissured rock during creep loading and unloading stages. The injected gas pressure was 3 MPa, and that of the outlet gas was 0.1 MPa. The permeability can be calculated using Eq. (1)^39^. The physical parameters of the specimens and experimental designs are presented in Tables 1 and 2.

1 $$k=\frac{Q\cdot \mu \cdot L\cdot 2{p}_{d}}{A\cdot ({p}_{u}^{2}-{p}_{d}^{2})}$$

where *Q* represents gas flow velocity (m^3^/s); *L* and *A* represent the length (m) and the cross-sectional area (m^2^) of the rock specimen, respectively; *μ* is the viscosity of nitrogen (Pa·s); and *P*_*u*_ and *P*_*d*_ represent the upstream and downstream gas pressure (MPa), respectively.

**Table 1:** Basic parameters of the tested red sandstone specimens in this research.

Specimen	*α*/°	*L*/mm	*D*/mm	*M*/g	*ρ*/kg/m^3^	*σ*_3_/MPa	*σ*_P_/MPa	TCS/MPa	Testing design
EW-1^#^	Intact	99.40	49.99	471.20	2415.25	30	271.82	301.82	Triaxial compression
ES-0-1^#^	0	100.50	49.99	478.10	2423.79	30	163.85	193.85	Triaxial compression
ES-15-1^#^	15	101.20	49.99	482.70	2430.19	30	159.75	189.75	Triaxial compression
ES-30-1^#^	30	99.60	49.99	472.90	2419.10	30	157.70	187.70	Triaxial compression
ES-45-1^#^	45	100.70	49.99	480.70	2432.14	30	168.16	198.16	Triaxial compression
ES-60-1^#^	60	99.50	49.99	475.10	2432.79	30	169.15	199.15	Triaxial compression
ES-75-1^#^	75	100.90	49.99	478.50	2416.21	30	178.30	208.30	Triaxial compression
ES-90-1^#^	90	99.90	49.99	468.40	2388.89	30	203.02	233.02	Triaxial compression
EW-10^#^	Intact	100.70	49.99	480.7	2432.14	30	—		Creep compression
ES-0-2^#^	0	101.40	49.99	485.80	2440.98	30	—		Creep compression
ES-15-2^#^	15	102.00	49.99	482.70	2414.63	30	—		Creep compression
ES-30-2^#^	30	100.20	49.99	474.70	2413.77	30	—		Creep compression
ES-45-6^#^	45	100.20	49.99	482.50	2453.43	30	—		Creep compression
ES-60-2^#^	60	99.50	49.99	477.50	2445.09	30	—		Creep compression
ES-75-2^#^	75	100.40	49.99	479.50	2433.32	30	—		Creep compression
ES-90-2^#^	90	100.00	49.99	478.60	2438.47	30	—		Creep compression

Note: *α*: fissure dip angle; *L*: length; *D*: diameter; *M*: mass; *ρ*: density; *σ*_3_: confining pressure; *σ*_p_: peak deviatoric stress; TCS = *σ*_3_ + *σ*_p_.

**Table 2:** Applied deviatoric stress levels in creep tests (*σ*_3_ = 30 MPa).

Specimen	*α*/°	*σ*_3_/MPa	S1	S2	S3	S4	S5	S6
EW-10^#^	Intact	30	180	205	220	240	260	274*
ES-0-2^#^	0	30	100	115	130	145	154.47*	
ES-15-2^#^	15	30	100	115	130	145*		
ES-30-2^#^	30	30	100	115	130	145*		
ES-45-6^#^	45	30	100	115	130	145*		
ES-60-2^#^	60	30	100	115	130	145	150.41*	
ES-75-2^#^	75	30	115	130	145	160	170	177.94*
ES-90-2^#^	90	30	130	145	160	175	193.07*	

Note: *Represents the final failed stress.

All the figure legends:

## Creep Behavior of Red Sandstone Containing a Single Fissure

**Figure 3:**
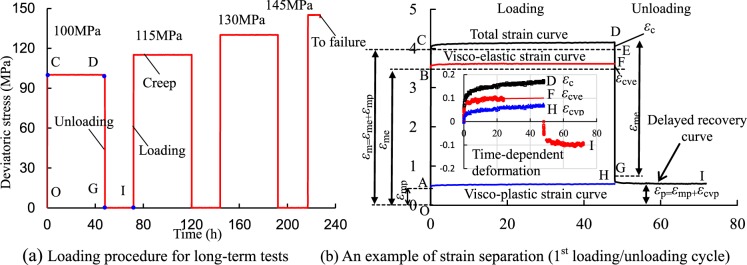
Loading procedure and a strain separation case (Fissured sandstone with 45° dip angle). Note: *ε*_m_: instantaneous strain; *ε*_me_: instantaneous elastic strain; *ε*_mp_: instantaneous plastic strain; *ε*_c_: creep strain; *ε*_cve_: visco-elastic strain; *ε*_cvp_: visco-plastic strain.

**Figure 4:**
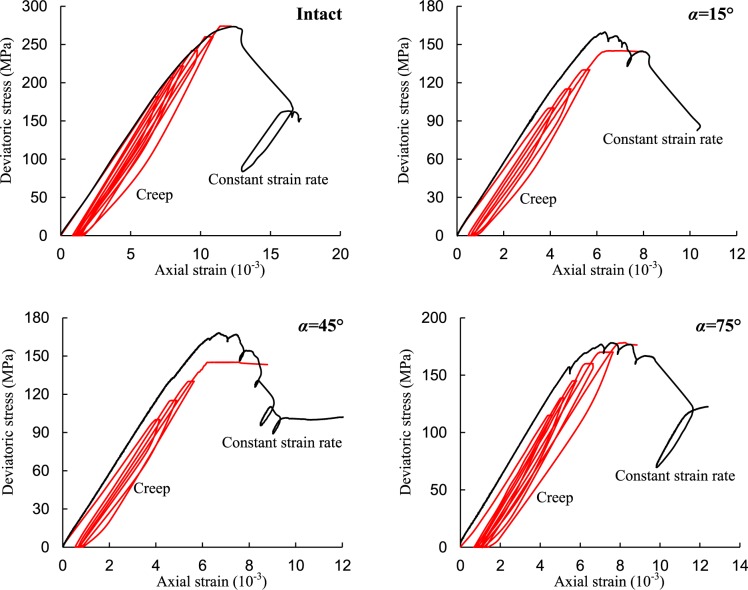
Typical short-term triaxial stress-strain curves of red sandstone (intact specimen, fissured specimens for α = 15°, 45°, and 75°). Note: the platforms on the creep curves mean creep deformations under constant stress levels.

**Figure 5:**
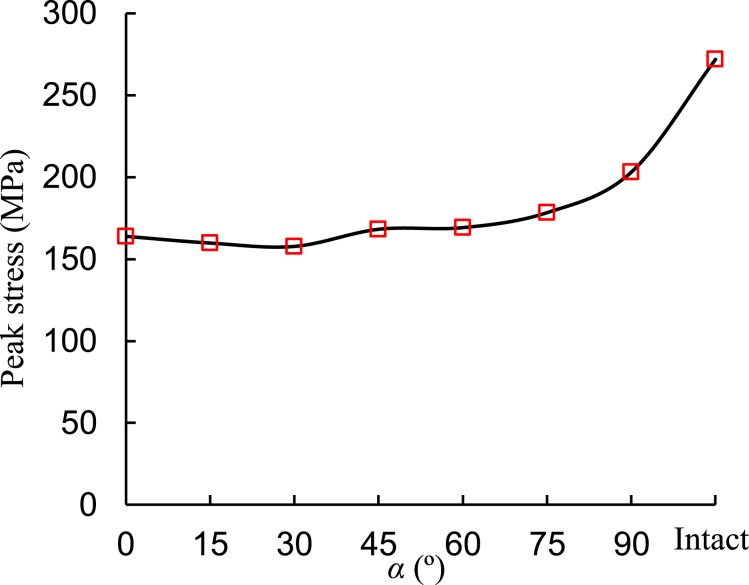
Relationship between fissure angle and peak stress.

Under compression, the rock specimen deforms gradually and different types of deformation compose the total deformation. When a constant loading is applied to the specimen, instant elastic and plastic deformation occurs, and visco-elastic and visco-plastic deformation occur with increasing time. When the loading is removed, the instant elastic deformation will be recovered soon and the visco-elastic deformation will recover gradually. The instant plastic and visco-plastic deformation cannot recover. Therefore, the elastic, viscous, and plastic deformations of the fissured rocks can be separated using the multi-step loading and unloading method. The creep loading-unloading path and an example of strain separation are plotted in Fig. 3. Finally, the influences of stress ratio (SR) and dip angle on the elastic-visco-plastic deformations of the single-fissured red sandstones can be quantitatively analyzed. The deviatoric stress-strain curves of the intact specimen and the fissured specimen are presented in Fig. 4 and Appendix 1. The relationship between fissure angle and peak stress is presented in Fig. 5. The peak stress of the intact specimen was nearly 1.3 times that of the 90° specimen, which can be subjected to more loading.

**Figure 6:**
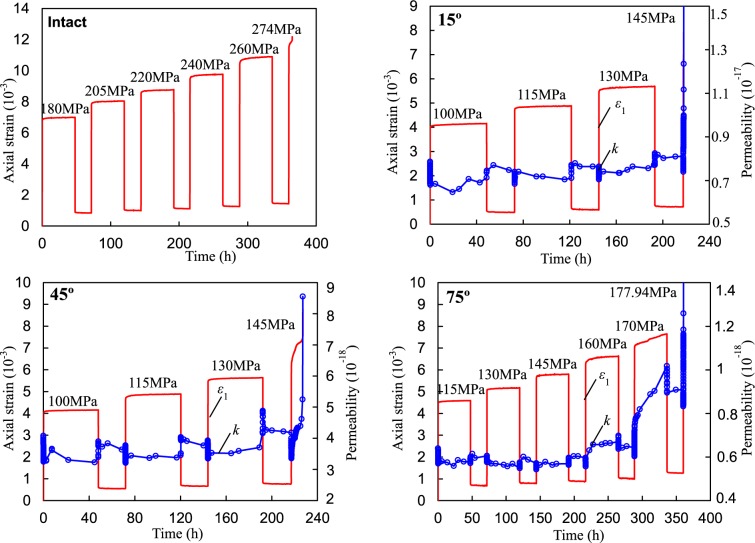
Typical axial strain, permeability vs. time curves of red sandstone (intact specimen, fissured specimens for α = 15°, 45°, and 75°).

The creep deformations of the intact and fissured rocks with various fissure dip angles under multi-step loading-unloading conditions are plotted in Fig. 6 and Appendix 2. Notably, the creep rate means that the strain rate evolves as time increases under constant loading during the total creep stage. The steady state denotes that the creep rate was almost constant or the creep rate increased approximately linearly during the creeping stage. It is clear that the axial deformation increased with increasing axial deviatoric stress and creep time under constant stresses. The irrecoverable axial deformation increased with the cyclic numbers of loading and unloading. Under relative low-stress ratios, the axial deformations mainly contained decelerating and steady-state creep deformations, whereas primary, secondary, and tertiary creep occurred under creep failure stress levels. Under unloading conditions (the deviatoric stress was 0 MPa), elastic-viscosity recoverable deformations were produced during this stage. For specimens with fissure dip angles of 0, 15, 30 and 75°, accelerating creep occurred in a very short time. The elastic, viscous, and plastic deformations of those specimens will be detailed in the following sections.

### Instantaneous elastic and plastic deformations

**Figure 7:**
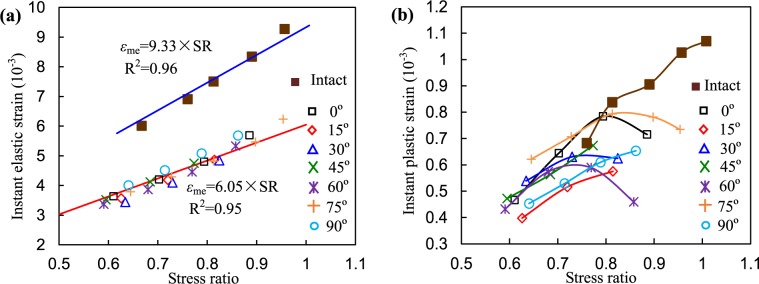
Relationships between stress ratio and instantaneous elastic and plastic strains of intact and fissured red sandstone.

Here, the ratio of creep deviatoric stress to peak deviatoric stress is termed as SR. The relationships between SR and instantaneous elastic of the intact and the single-fissured red sandstones with various dip angles are presented in Fig. 7. It can be seen that the instantaneous elastic strains of the specimens were linearly corrected to the stress ratio when SR varied from approximately 0.6 to 0.95, as shown in Fig. 7a. Two linear equations can describe the correlations between instantaneous elastic strain and SR of the intact and fissured specimens. Although the SRs applied on the intact specimen were similar to those of the fissured specimens, the former had relatively larger instantaneous elastic strain than the latter. The relationships between SR and the instant plastic strain of the fissured sandstones are different (Fig. 7b). For the intact specimen and the fissured specimens with 45°, 75°, and 90° dip angles, the instant plastic strains increased approximately linearly as SR increased. However, for the other specimens, similar results were observed under low SRs, whereas instant plastic strain decreased under high SR.

### Visco-elastic and visco-plastic deformations

Both visco-elastic and visco-plastic deformations of rock are important parts of the long-term time-dependent deformation of rock, which quantitatively reflect the time-based deformation properties of rock. To better investigate the relationship between deformation and time during creep stage, it is necessary to separate the recoverable and irrecoverable deformations as time goes by. Therefore, the visco-elastic and visco-plastic deformations of the fissured red sandstone rock were separated using a multi-step loading and unloading method as shown in Fig. 3b.

**Figure 8:**
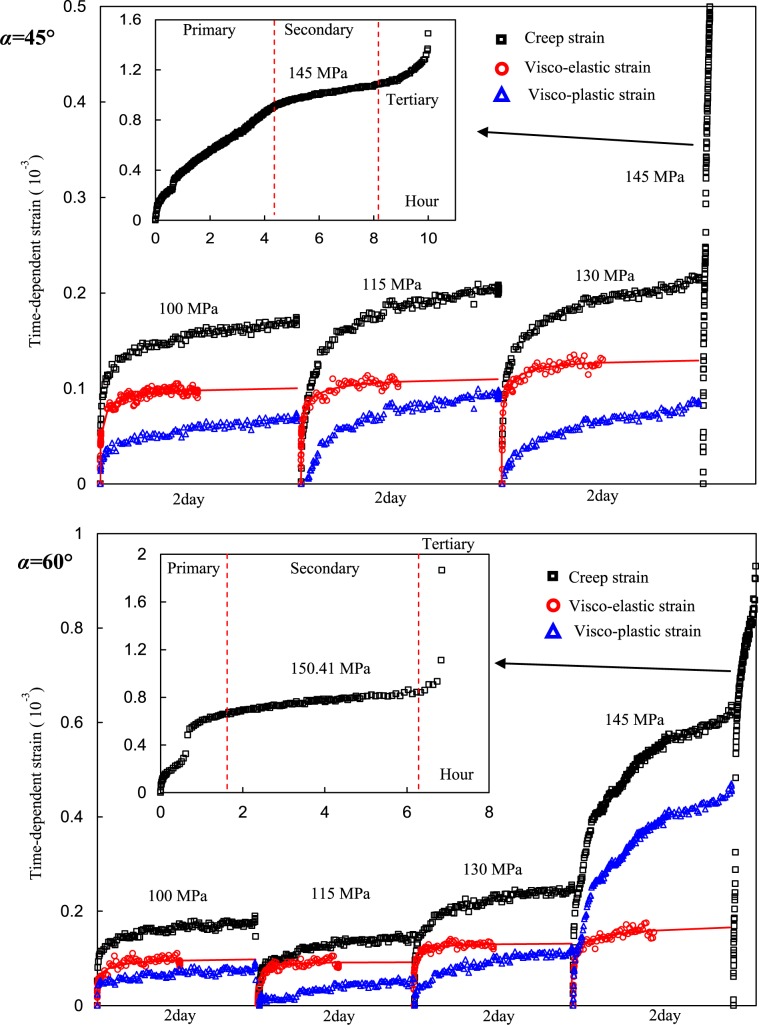
Creep strain, visco-elastic strain and visco-plastic strain of the fissured red sandstone.

Typical separation results of the fissured rock with 45° and 60° inclinations are plotted in Fig. 8. The fitting method of the visco-elastic strain curves was obtained according to the report by Yang and Hu^37^. The creep strains, visco-elastic strains, and visco-plastic strains of the 45° specimen increased with increasing stress steps. Under deviatoric stress of 145 MPa, it showed primary, secondary and tertiary creep lasting approximately 10 h. The creep strain of the 60° specimen first slightly decreased when deviatoric stress increased from 100 to 115 MPa. After that, it increased gradually with increasing deviatoric stress. The visco-plastic strain showed a similar evolution. The visco-elastic strain had no obvious variation when deviatoric stress was 100 and 115 MPa but increased with increasing stress levels. Generally, the time-dependent strains increased with increasing deviatoric stress.

**Figure 9:**
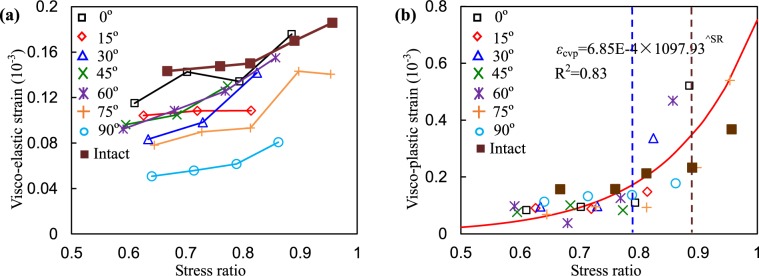
Relationships between visco-elastic and visco-plastic strains and stress ratio of the specimens.

Figure 9 plots the evolution of the visco-elastic and visco-plastic strains vs. SR. The visco-elastic strains of the specimens had approximately linear correlation with SR. The specimen with *α* = 90° fissure had the minimum visco-elastic strain, but the visco-elastic deformation of the specimen with *α* = 0° fissure was larger than of the other specimens. In contrast, the visco-elastic strain of the intact specimen was higher than those of the fissured specimens. However, the visco-plastic strains of the specimens nonlinearly increased with SR as shown in Fig. 9b, whose relationship can be described by a nonlinear fitting equation. It is also clear that the visco-plastic strains changed not significantly when SR was less (approximately 0.8).

**Figure 10:**
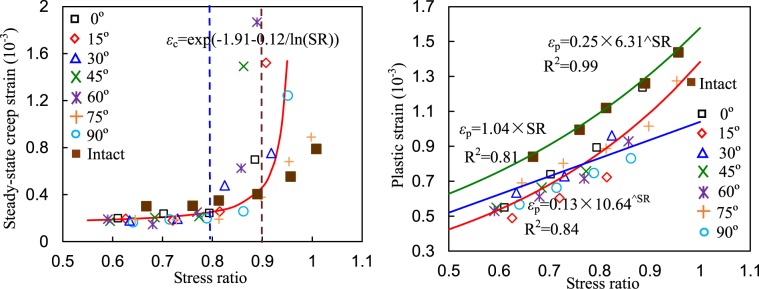
Relationships between creep strain and plastic strain and stress ratio.

The relationship between the creep and total plastic strains of the specimens and the SR are shown in Fig. 10. In Fig. 10a, the creep strain increased nonlinearly with SR, which can be described by a fitting equation. Specifically, when SR was less than approximately 0.8, the creep slightly varied but significantly increased beyond that critical point. The plastic strains increased with SR, whose fitting equations are presented in Fig. 10b, in which the nonlinear fitting equation shows a larger R^2^ value than the linear fitting result. For intact specimens, the plastic strain also had a nonlinear correlation with SR. In contrast, the plastic strains of the intact specimen were larger than those of the fissured specimens.

### Creep rate

**Figure 11:**
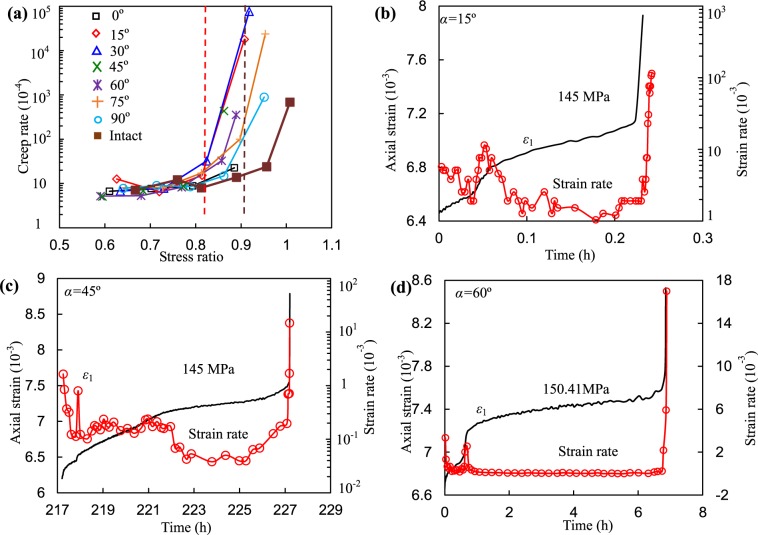
Relationship between creep rate and stress ratio and the evolution of strain rate during creep.

The evolution of the strain rate during the creep stage is essential to analyze the time-based deformation of rock. Hao *et al*.^29,40^ presented a detailed study and discussion about the creep rate and how to define the steady-state and provided a relationship between failure-time with steady-state creep rate. Considering that the magnitude of the creep rates under relative high deviatoric stresses was larger than those under low-stress levels, the creep rates were plotted in logarithmic coordinates. In Fig. 11a, the steady-state creep rates (strain rates during the secondary creep stage) increased with deviatoric stress. When SR was less than approximately 0.8 (0.9), the steady-state creep rates had no distinct variations but suddenly increased beyond the SR point for fissured (intact) specimens. In contrast, the rates of the intact specimen were relatively lower than those of the fissured rock specimens. Because of space limitations, the evolutions of strain rate during primary, secondary, and tertiary creep stage of the specimens with a fissure dip angle of 15°, 45°, and 60° were presented, as shown in Fig. 11b–d. The strain rate reflected the evolution of an axial strain of rock during the creep stage, showing decreasing trend during primary and secondary creep stages and showing increasing trend at accelerating creep stage.

### Effect of fissure dip angle on the deformations

**Figure 12:**
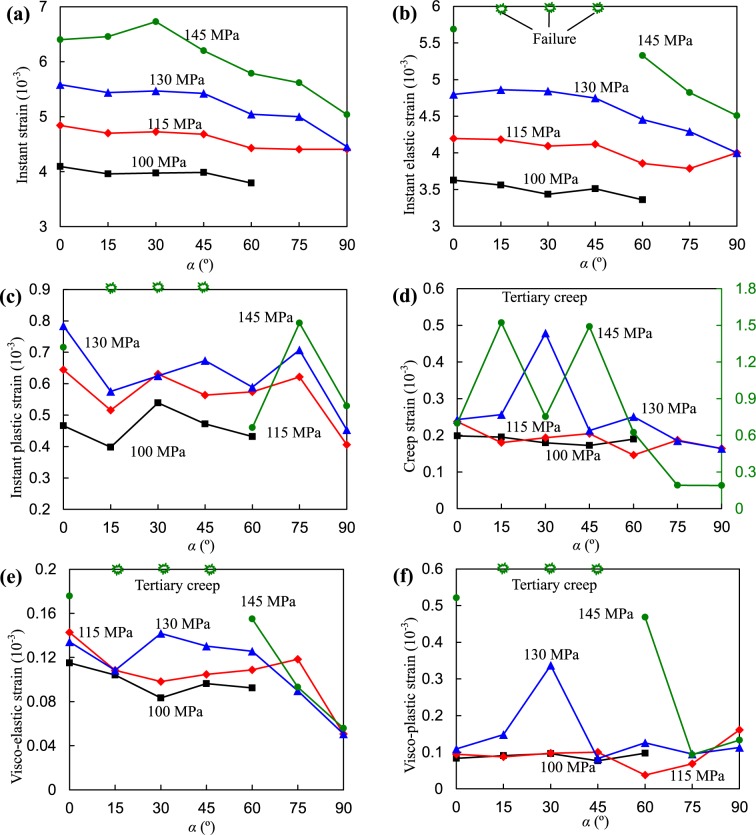
Influence of fissure dip angle on the elastic, viscous and plastic deformations of the specimens.

As the previous results mentioned above, the pre-existing fissure inclination had a visible effect on the deformation behavior of the rock. Specifically, the fissure dip angle affected the elastic and plastic strains and visco-elastic and visco-plastic strains of the rock specimens. It is evident in Fig. 12a that the instantaneous strains under deviatoric stresses of 100, 115, and 130 MPa had no obvious variations when *α* was less than 45° but decreased gradually with increasing dip angle. However, the instantaneous strain first increased as *α* increased from 0 to 30° and then decreased with an increasing dip angle under a deviatoric stress of 145 MPa. The higher the deviatoric stress, the larger the instantaneous strain.

Figure 12b illustrates the variations in the instant elastic strains of the specimens vs. fissure dip angle. It seems that the downtrend of the instantaneous elastic strain became smaller as the deviatoric stress level increased when *α* was less than 45°. Beyond 45°, the instant elastic strains notably decreased. Because the specimens with 15°, 30°, and 45° fissure angles showed creep failure under stress level of 145 MPa, instantaneous elastic strains were unable to be separated. It seems that *α* had no obvious influence on the instantaneous plastic strains, creep strains, and visco-plastic strains, as shown in Fig. 12c,d,f. The visco-elastic strain seems to decrease with increasing dip angle.

## Damage and Permeability Evolution Behavior of Red Sandstone Containing a Single Fissure

### Damage evolution behavior of red sandstone containing a single fissure

To characterize the state properties of materials, the damage concept was firstly proposed by Kachanov^41^. Damage variable (*D*) can quantitatively describe the deterioration degree of a material under changing stress state. However, it is difficult to measure *D* directly. Therefore, *D* is calculated using other physical or mechanical parameters of materials, such as elastic modulus, ultrasonic wave velocity, density and severity, energy, strain, and acoustic emission accumulates^42^. However, one vital principle to definite damage variables is that it should be easily measured to build a relationship with the macroscopic mechanical state. Materials undergoing elastic strain and visco-elastic strains can be recovered after unloading, but those undergoing visco-plastic and plastic strains cannot. Hence, the damage variable of the rock under cyclic loading-unloading conditions can be calculated using the ratio of non-elastic strain (instant plastic strain and visco-plastic strain), as shown in Eq. (2). Finally, *D* can be calculated by instantaneous elastic and visco-elastic strains.

2 $$D=1-\frac{{\varepsilon }^{e}}{\varepsilon }=1-\frac{{\varepsilon }_{me}+{\varepsilon }_{cve}}{{\varepsilon }_{m}+{\varepsilon }_{c}}=\frac{{\varepsilon }_{mp}+{\varepsilon }_{cvp}}{{\varepsilon }_{m}+{\varepsilon }_{c}}$$

where *ε*^e^ and *ε* represent the elastic strain and the total strain, respectivly; *ε*_me_ and *ε*_cve_ represent the instantaneous elastic strain and the visco-elastic strain, respectivly; and *ε*_m_ and *ε*_c_ represent the instantaneous strain and the creep strain of the specimen, respectivly.

**Figure 13:**
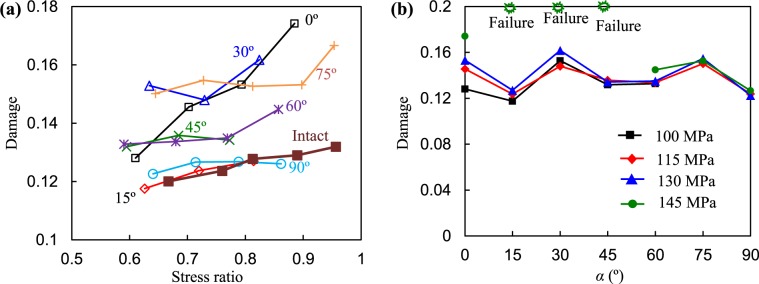
Relationships between damage and stress ratio and dip angle.

The results of *D* under different SRs are plotted in Fig. 13. From Fig. 13a, *D* increased with increasing axial SR generally. The damage of the intact specimen seemed relatively small. At identical SRs, the degree of damage to the rock specimen had no obvious relationship with the dip angle, as shown in Fig. 13b. It seems that the higher the stress level, the more serious the damage.

### LTS of red sandstone containing a single fissure

LTS is an important index to predict rock failure and for design engineering. This parameter is determined mainly by direct and indirect methods. Schmidtke and Lajtai^43^ reported that the minimum creep failure stress of granite under uniaxial compression was 60% of its uniaxial compressive strength. Szczepanik *et al*.^44^ found that dilatancy occurred when the loading was 70–80% of the uniaxial compressive strength, and this stress point can be regarded as its LTS. Generally, the determining method can be classified as (1) unsteady deformation method^45^; (2) the stress threshold method (*σ*_cd_)^46,47^; and (3) the isochronal stress-strain curves method (ISSCM) according to the creep experimental results^48–50^.

**Figure 14:**
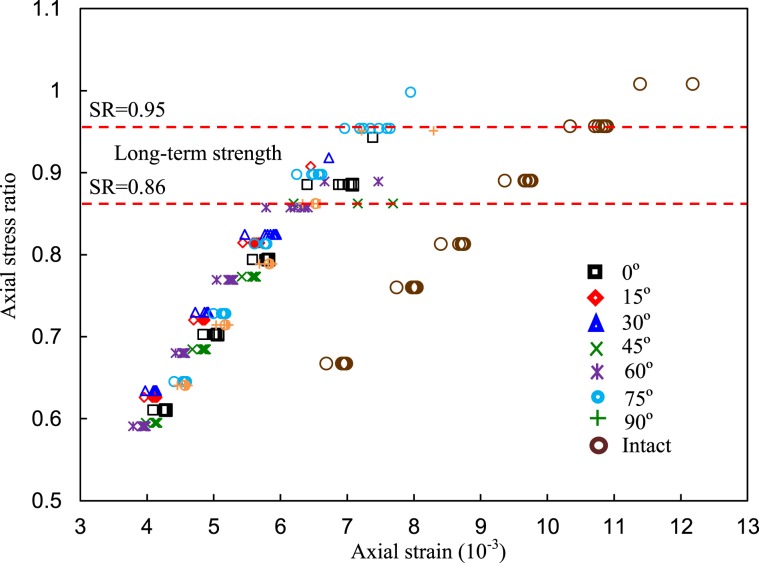
Isochronal stress ratio-strain curves of the specimens.

**Figure 15:**
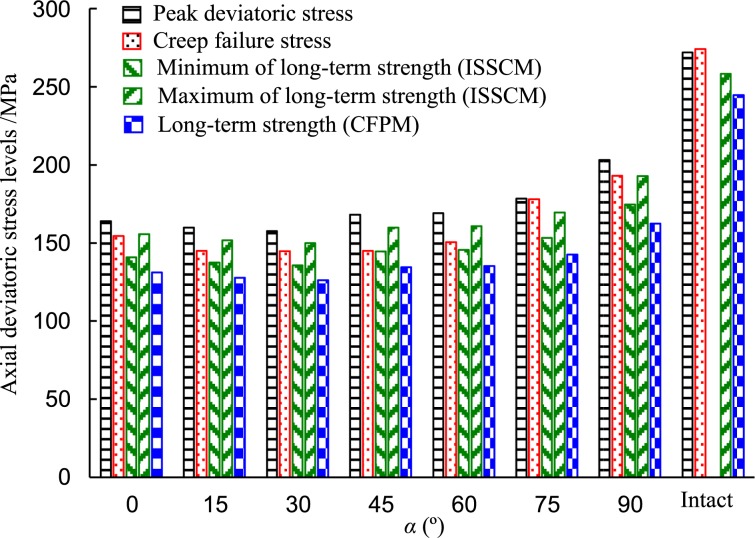
Characteristic stresses of the red sandstone.

To better understand the influence of stress level on the long-term strength, the isochronal stress ratio-strain curves of the intact specimen and fissured rock with various fissure dip angles are plotted in Fig. 14. When SR was less than 0.86, the isochronal curves were linear. However, when SR was between 0.86 and 0.95, the axial strains significantly increased and deviated from the original linear relationships. Hence, the LTS of the red sandstone with a single fissure was the corresponding stress level at the SR range from 0.86 to 0.95 (Red dotted line in Fig. 14). In contrast, the isochronal stress-strain curves of the intact specimen were located to the right of those of the fissured specimens, showing much more apparent deformation. The turning point of the curve of the intact specimen was approximately 0.95. Moreover, according to the previous results of this study, the steady-state creep rates, visco-plastic strains, and the creep strains of the fissured red sandstones significantly increased when SR was larger than 0.8. These phenomena also suggested that SR = 0.8 can be regarded as the lifetime of the red sandstone containing a single pre-existing fissure and SR = 0.9 for the intact specimen. This method can be termed as ‘critical feature point method’ (CFPM). However, for the intact specimen, the LTS was larger than those of the fissured specimen. Finally, a series of characteristic stresses of the fissured red sandstone containing different fissure dip angles are classified in Fig. 15.

Under *σ*_3_ = 30 MPa condition, the failure stress under constant strain rate loading was larger than that at creep compression. Fissure dip angle had a great influence on the failure stress of the fissured rock. Both the peak deviatoric stress and creep failure stress slightly decreased when the fissure dip angle increased from 0 to 30° and they had no obvious changes when the dip angle was 30° and 45°. However, the failure stresses increased significantly as the dip angle increased from 60° to 90°. The intact red sandstone had the highest failure stress, creep failure stress, and LTS compared to those of the fissure specimens. Therefore, it is clear that the fissure dip angle had a significant influence on the failure stresses on the fissured rock.

### Creep failure behavior of red sandstone containing a single fissure

**Figure 16:**
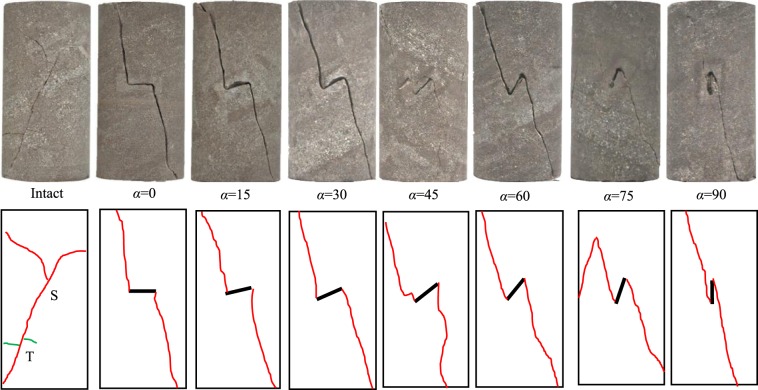
Short-term failure patterns of the intact and fissured red sandstone specimens (*σ*_3_ = 30 MPa).

**Figure 17:**
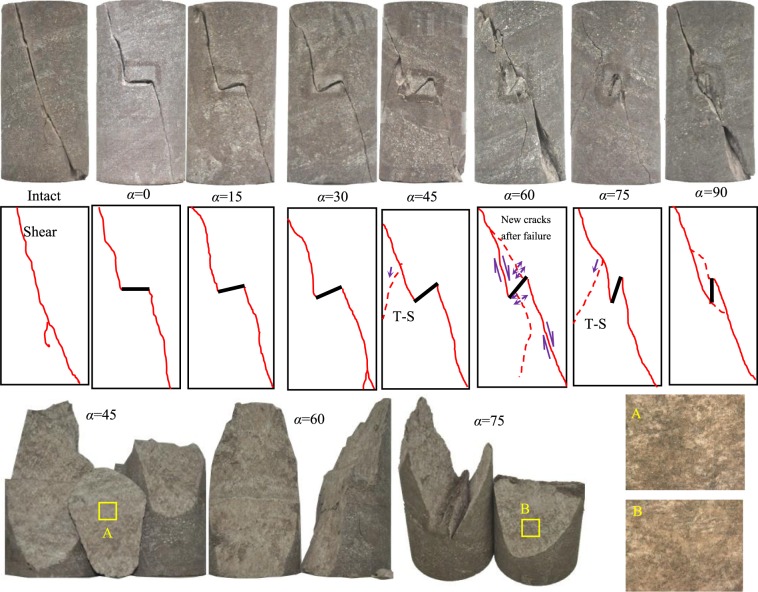
Creep failure patterns of the intact and fissured red sandstone specimens (*σ*_3_ = 30 MPa).

The triaxial compression and creep failure modes of the intact and fissured red sandstones containing a single fissure are presented in Figs. 16 and 17. In Fig. 16, shear failure occurred in the intact specimen. A macro shear crack and two small tensile cracks appeared. The tensile cracks were induced by the bending moment when macro shear sliding occurred along the shear surface. The fissured specimens commonly showed shear anti-wing cracks. Figure 17 shows that the cracking patterns of the specimens are similar. Macro shear anti-wing cracks occurred at the pre-existing fissure tips. The anti-wing cracks connected the upper left and bottom right of the specimen and the tips of the pre-existing fissures. For specimens with a dip angle of 45° and 75°, tensile-shear cracks appeared on the left side of the pre-existing fissure. These two cracks were induced by the bending moment when shear anti-wing crack sliding occurred along the shear plane. Then, under the restriction of the confining pressure, shear friction occurred along the crack surface, showing a scratch on the surface, as shown at positions A and B. For the specimen containing a fissure dip angle of 60°, axial loading was continually applied on the specimen after creep failure until new static stress was reached. After that, the axial deformation continued to increase. During this loading and short-term creep, new cracks appeared. The interpretation is that the pre-existing unfilled fissure closed after tertiary creep occurred and then subjected new stress until further creep stress occurred. During this bearing process, tensile strain concentration occurred at the ends of the closed tip and tensile force induced tensile cracks after creep failure. However, because of the confinement of the confining pressure, the generated tensile cracks could not be opened, so they were closed and shear sliding occurred. Finally, the tensile-shear failure model was formed. However, for the specimen with a 90° dip angle, the shear strain concentration mainly occurred around the tips of the pre-existing fissure. Therefore, shear cracks around the tips of the fissure appeared and connected the macro shear crack. In contrast, the intact specimen showed a continuous shear fracture. Therefore, it is clear that the pre-existing fissure had a considerable influence on the creep failure model of the specimen.

### Permeability evolution behavior of red sandstone containing a single fissure

**Figure 18:**
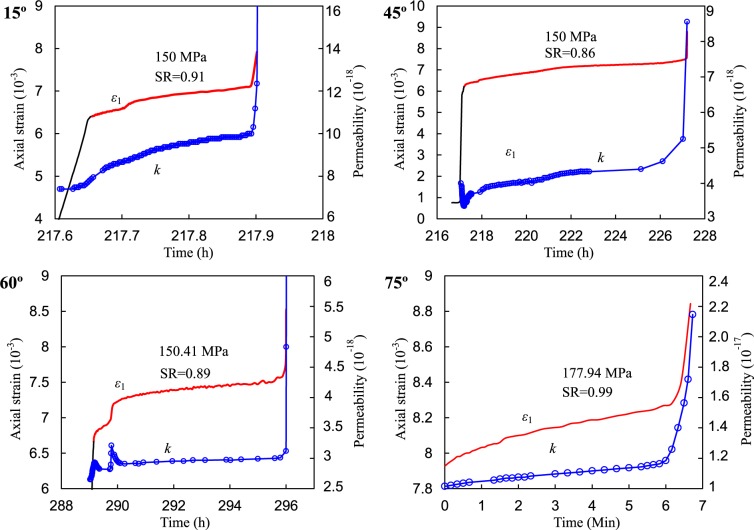
Permeability evolutions during three-stage creep progress.

During the long-term deformation process, the gas permeability evolution of the red sandstones containing a single pre-existing fissure with different dip angles are presented in Fig. 18 and Appendix 2. Clearly, the permeability varied with deviatoric stress and creep time. When the deviatoric stresses were applied and removed, the permeability immediately decreased and increased, respectively. Moreover, the permeability at unloading stages (visco-elastic recovery stage) was slightly more significant than that at loading stages (creep stage under deviatoric stress compression). The reason is that the specimens were compressed under different deviatoric stress levels, so the seepage channels were relative narrower than those at the hydrostatic state. Therefore, the corresponding permeability was relatively small. Generally, the permeability first decreased slightly and finally exhibited a sudden rise when the tertiary creep occurred during the multi-step loading and unloading process. Liu *et al*.^51^ reported that the permeability of Cox claystone showed similar evolution, but it had no variations when the deviatoric stress was applied under multi-step creep conditions.

Moreover, the permeability evolutions in the fissured red sandstone during the three-stage creep process were distinct. Generally, permeability increased with increasing axial strain, especially during accelerating creep stage. This suggests that the variation in permeability is not only governed by loading but is also influenced by the deformation. For specimens with fissure dip angles of 15°, 45°, and 75°, the permeability slightly increased during primary and secondary creep stage but significantly increased during the accelerating creep stage. However, for the specimen with 60° fissure dip angle, the permeability increased when applied creep loading. When the specimen started exhibiting primary creep, the permeability slightly decreased with time. The deformation suddenly increased and then increased at a decreasing rate during the primary creep stage. Correspondingly, the permeability also had an increment and then decreased with time. The result suggests that the permeability significantly depends on the rock deformation. When the deviatoric stress was applied, the seepage channels in the rock specimen are opened, so the permeability increased. However, the creep deformation narrowed the opened channels, thus decreasing the permeation.

## Discussion

### Influence of pre-existing fissure

**Figure 19:**
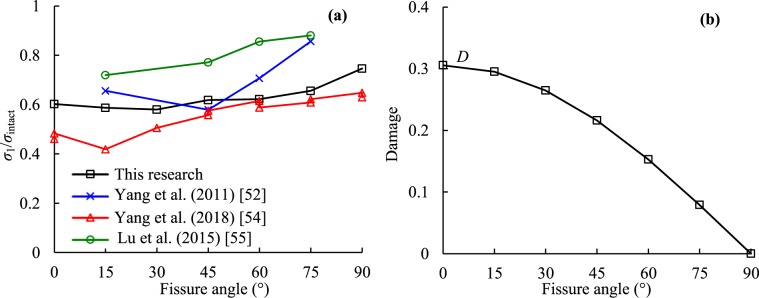
Influence of fissure angle on normalized strength (*σ*_1_/*σ*_intact_) of sandstone.

As reported previously, the strength of the sandstone can be decreased by pre-existing fissures compared with that of intact sandstones^52–55^. This is because of stress consecration at the tips of the pre-existing fissure and macro cracks formed easily than in the no pre-existing flaw sandstone. The normalized peak strength expressed by the ratio of the peak strength of the fissured sandstone to that of the intact sandstone increased as flaw angle increased as presented in Fig. 19a. From the view of damage mechanics, the pre-existing fissure can be viewed as initial damage. Kachanov^41^ defined simple damage from the view of the principle of effective stress based on the actual loading area of the material as shown in Eq. (3). The variation in the damage variable affected by the fissure angle is presented in Fig. 19b. During creep experiments, although the fissured sandstones were subjected to identical stresses, the effective stress in each specimen with different fissure angle was different. Higher effective stress was generated for lower flaw angles. As a result, the fissured sandstone with a lower flaw angle showed larger deformation than that in the specimen with a higher fissure angle.

3 $$D=\frac{{A}_{D}}{A}=1-\frac{{A}_{e}}{A},\,{\sigma }^{e}=\frac{\sigma }{(1-D)}$$

where, *A* is the cross-section area of the material (A = 1962.71mm^2^), *A*_D_ and *A*_e_ are the damaged and effective areas of the material under loading, respectively; and *σ* and *σ*_e_ represent the applied stress and the effective stress, respectively.

### Influence of loading/unloading cycle

In underground coal mining and tunnel excavations, rocks are usually subjected to loading/unloading cycles^37,56,57^. The long-term mechanical behaviors of the rocks under this loading condition are essential for designing and evaluating the long-term stability for engineering applications. The creep behavior is related to the fatigue effect^58^. Moreover, subcritical crack growth induced time-dependent damage plays a vital role until rupture^59,60^.

**Figure 20:**
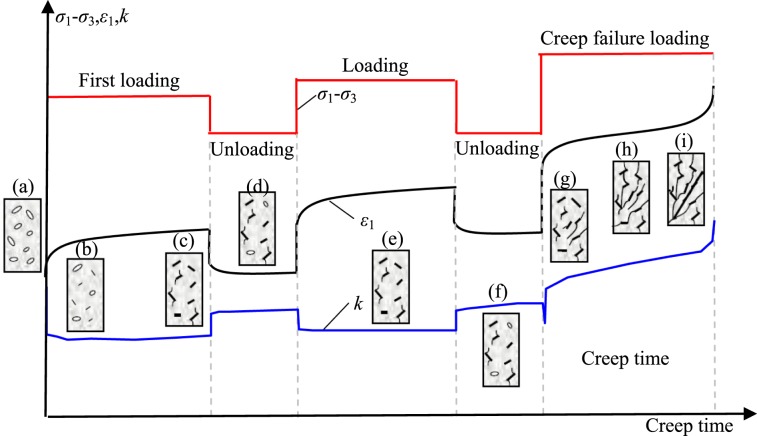
A schematic diagram of the deviatoric stress, axial strain and permeability during loading/unloading cycles (after Xu and Yang^61^).

Sandstone is a clastic particle aggregate. The microcracks are distributed randomly and not all are entirely closed under hydrostatic stress state (Fig. 20a). When deviatoric stress is applied, the horizontal microcracks are closed, and most of them can not be reopened. Consequently, the permeability reduces significantly (Fig. 20b). Mineral grain friction and micro-cracks are further closed under constant loading, and the specimens are deformed as time increases (Fig. 20c), whereas the permeability showed no distinct reduction. During the unloading state, when deviatoric stress was removed, the compacted grains were recovered, instant elastic deformation was released and visco-elastic deformation was also released with time, but some parts of irrecoverable deformation (instant plastic deformation and visco-plastic deformation) accumulated. Parts of horizontal cracks connected with vertical cracks were reopened, so the permeability also increased (Fig. 20d). With increasing loading/unloading cycles, the microcracks showed a cyclic close-reopen stage (Fig. 20e–f). The accumulated irrecoverable deformation increased. Microcrack growth increased the crack density and crack length under constant loading. For fissured sandstones, local stress concentration and damage occurred at the tips of the pre-existing fissures. If the applied stresses generated a higher stress intensity factor than the fracture toughness, creep rupture appeared. Thus, the LTS of the fissured and intact sandstones under creep loading was less than its peak strength, and the permeability increased significantly (Fig. 20g,i).

Most of the previous investigations were performed for triaxial creep on intact rock specimens. However, this study presents a series of experimental results of the sandstone with pre-existing fissure under cyclic loading/unloading conditions. The results suggest that flaw significantly reduces the LTS of the sandstone. Cyclic loading/unloading increases the stress-induced damage and irrecoverable deformation. This study may provide a reference for geoscience research and energy resources.

## Conclusions

In this study, the elastic-viscoplastic deformation behaviors, LTS and gas permeability evolution during creep of intact and fissured red sandstones were investigated. Fissured specimens containing a single fissure with various fissure dip angles (0 to 90°, interval 15°) were produced first. Then, constant strain rate loading experiments and cyclic loading and unloading creep tests under a confinement of 30 MPa were conducted on the specimens. The conclusions obtained from the experimental results are listed as follows.

1. The irrecoverable axial deformation accumulated as the cyclic numbers of loading/unloading increased. The instantaneous elastic deformation had a linear correlation with SR. The visco-elastic deformation and the viscoplastic deformation had linear and nonlinear relationships with SR, respectively. When SRå 0.8 (fissured specimen) and 0.9 (intact specimen), the creep strains and the steady-state creep rates nonlinearly increased with stress ratio; two nonlinear fitting equations can describe the relationships between total plastic deformation and stress ratio.
2. The instantaneous deformation, instant elastic deformations, and the visco-elastic deformations of the fissured rock decreased with fissure dip angle, especially when the dip angle was larger than 45°. However, the dip angle had no obvious influence on the instant plastic, visco-plastic and creep deformations.
3. The damage variable defined by the elastic deformation increased approximately linearly with SR, but the fissure dip angle had no obvious effects. The long-term strength of the intact (LTS ≤ 0.95) and fissured sandstone (0.86 ≤ LTS ≤ 0.95) determined according to isochronal stress-strain curves was larger than that determined by the presented CFPM (LTS ≤ 0.9, LTS ≤ 0.8). The creep failure modes were mainly shear along anti-wing cracks for the fissured specimens, but the intact specimen showed a shear fracture.
4. The permeability of the fissured sandstone showed stress and deformation dependence, decreasing with increasing loading and increasing with decreasing unloading and changing with time. During multi-step loading/unloading creep cycles, it first decreased and then showed a sudden rise when tertiary creep occurred. Moreover, the permeability increased slightly during primary and secondary creep stage but increased significantly during the accelerating creep stage under high SRs. However, it gradually decreased during primary and secondary creep under low SR.

## Supplementary information

Supplementary Appendix 1

Supplementary Appendix 2

Supplementary Appendix 3
