# Supplementary material for: Creep and permeability evolution behavior of red sandstone containing a single fissure under a confining pressure of 30 MPa
Source: Sci Rep. 2020 Feb 5;10:1900. doi: 10.1038/s41598-020-58595-2 (PMC7002402; doi:10.1038/s41598-020-58595-2)
Supplement: Supplementary file 1 — Supplementary Appendix 1 [file 41598_2020_58595_MOESM1_ESM.pdf]

Scientific Reports (2nd Revision)

Creep and permeability evolution behavior of red sandstone containing a single fissure under a confining pressure of 30 MPa

Sheng-Qi Yang*, Bo Hu

State Key Laboratory for Geomechanics and Deep Underground Engineering, School of Mechanics and Civil Engineering, China University of Mining and Technology, Xuzhou 221116, PR China;

*** Corresponding author:** Dr. Professor. Sheng-Qi Yang

Tel: +86-516-83995856

Fax: +86-516-83995678

E-mail address: yangsqi@hotmail.com

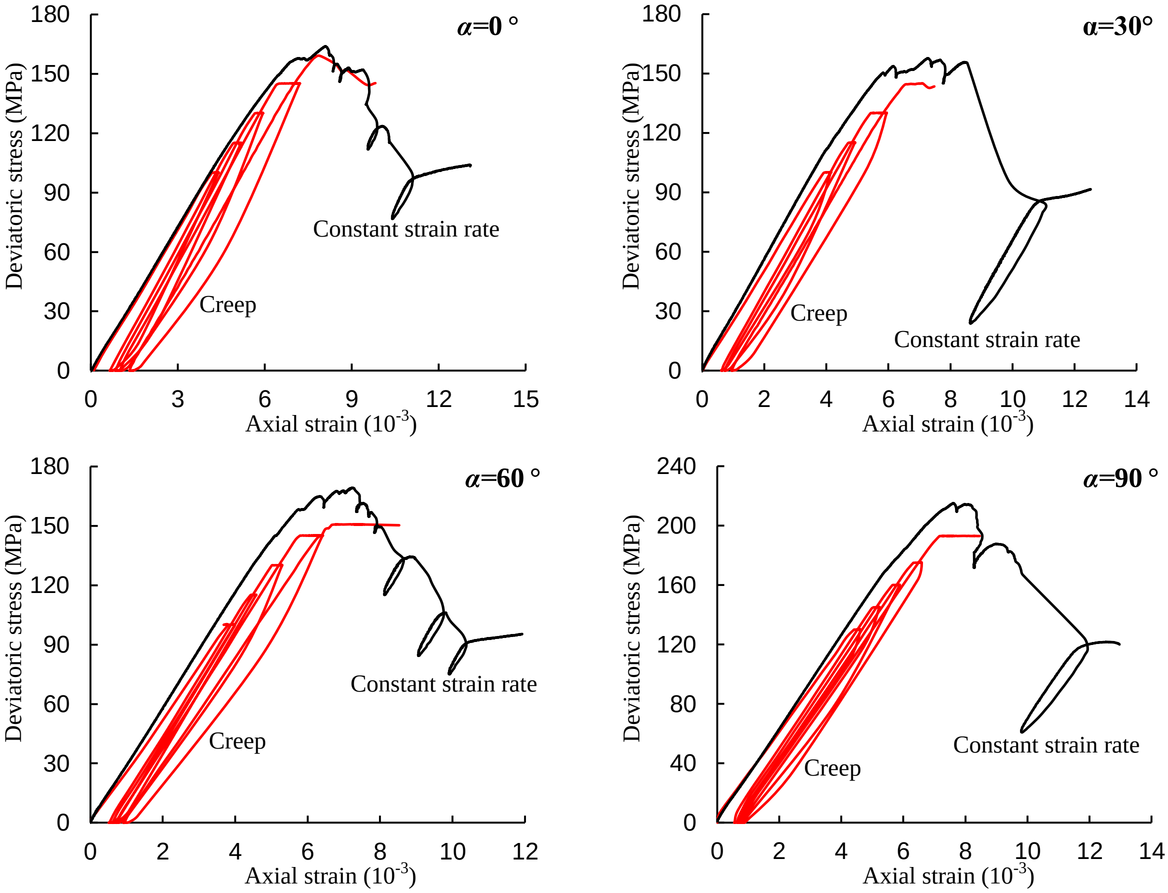

Appendix 1: Typical short-term triaxial stress-strain curves of fissured red sandstone (α=0 °, 30 °, 60 ° and 90 °). Note: the platforms on the creep curves mean creep deformations under constant stress levels.
