# Supplementary material for: Creep and permeability evolution behavior of red sandstone containing a single fissure under a confining pressure of 30 MPa
Source: Sci Rep. 2020 Feb 5;10:1900. doi: 10.1038/s41598-020-58595-2 (PMC7002402; doi:10.1038/s41598-020-58595-2)
Supplement: Supplementary file 2 — Supplementary Appendix 2 [file 41598_2020_58595_MOESM2_ESM.pdf]

Scientific Reports (2nd Revision)

Creep and permeability evolution behavior of red sandstone containing a single fissure under a confining pressure of 30 MPa

Sheng-Qi Yang*, Bo Hu

State Key Laboratory for Geomechanics and Deep Underground Engineering, School of Mechanics and Civil Engineering, China University of Mining and Technology, Xuzhou 221116, PR China;

*** Corresponding author:** Dr. Professor. Sheng-Qi Yang

Tel: +86-516-83995856

Fax: +86-516-83995678

E-mail address: yangsqi@hotmail.com

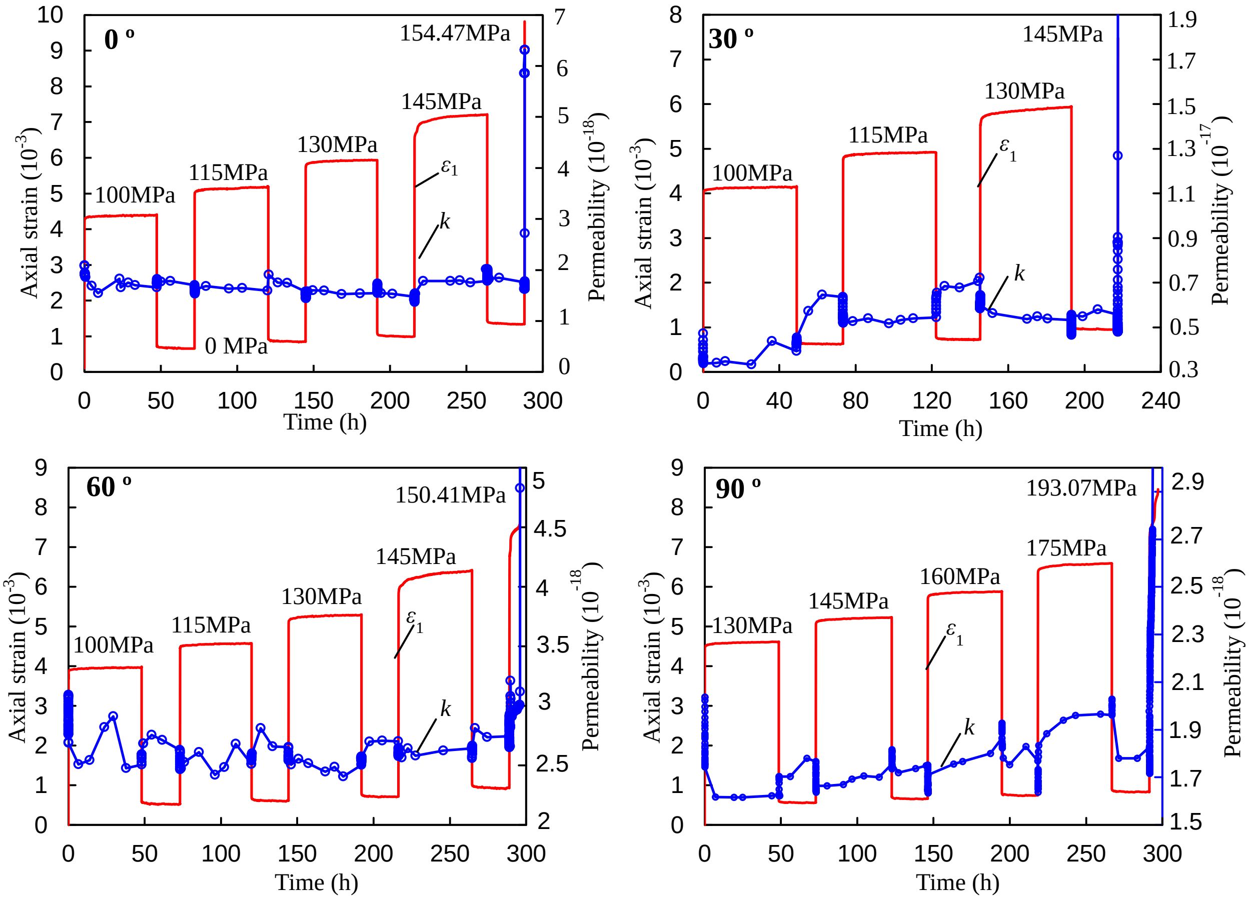

Appendix 2: Typical axial strain, permeability vs. time curves of fissured red sandstone (α=0 °, 30 °, 60 °and 90 °)
