# Supplementary material for: Creep and permeability evolution behavior of red sandstone containing a single fissure under a confining pressure of 30 MPa
Source: Sci Rep. 2020 Feb 5;10:1900. doi: 10.1038/s41598-020-58595-2 (PMC7002402; doi:10.1038/s41598-020-58595-2)
Supplement: Supplementary file 3 — Supplementary Appendix 3 [file 41598_2020_58595_MOESM3_ESM.pdf]

Scientific Reports (2nd Revision)

Creep and permeability evolution behavior of red sandstone containing a single fissure under a confining pressure of 30 MPa

Sheng-Qi Yang*, Bo Hu

State Key Laboratory for Geomechanics and Deep Underground Engineering, School of Mechanics and Civil Engineering, China University of Mining and Technology, Xuzhou 221116, PR China;

*** Corresponding author:** Dr. Professor. Sheng-Qi Yang

Tel: +86-516-83995856

Fax: +86-516-83995678

E-mail address: yangsqi@hotmail.com

Appendix 3: Explanation of the fitting procedure of the fitting curves in figures.

The fitting curves were produced in the Microsoft-Excel application.
Step 1: Select the X-axis data and Y-axis data in Excel and make a scatter chart;
Step 2: Click the data in the scatter chart and right-click the mouse;
Step 3: Find the “add Trendline” option and left click, select an appropriate or custom trendline;
Step 4 Also select “Display Equation on chart” and “Display R-squared value on chart”
